# A novel cgMLST for genomic surveillance of *Yersinia enterocolitica* infections in France allowed the detection and investigation of outbreaks in 2017–2021

**DOI:** 10.1128/spectrum.00504-24

**Published:** 2024-04-23

**Authors:** Anne-Sophie Le Guern, Cyril Savin, Fanny Chereau, Sabrina Tessier, Julien Guglielmini, Sylvie Brémont, Javier Pizarro-Cerdá

**Affiliations:** 1 Institut Pasteur, Université de Paris Cité, Yersinia Research Unit, Yersinia National Reference Laboratory, WHO Collaborating Centre for Plague Fra-140, Paris, France; 2 Santé publique France, Infectious Diseases Division, Saint-Maurice, France; 3 Santé publique France, Regions Division, Bourgogne-Franche-Comté Office, Dijon, France; 4 Institut Pasteur, Université de Paris Cité, Bioinformatics and Biostatistic Hub, Paris, France; University Paris-Saclay, Clamart, France

**Keywords:** *Yersinia enterocolitica*, enteric yersiniosis, cgMLST, outbreak, epidemiological investigation

## Abstract

Enteric yersiniosis, the third most common food-borne zoonosis in Europe, is mainly caused by the pathogen *Yersinia enterocolitica*. In France*,* the yersiniosis microbiological surveillance is conducted at the *Yersinia* National Reference Laboratory (YNRL). Since 2017, isolates have been characterized by whole genome sequencing (WGS) followed by a 500-gene *Yersinia*-cgMLST. We report here the data of the WGS-based surveillance on *Y. enterocolitica* isolates for the 2017–2021 period. The YNRL characterized 7,642 *Y. enterocolitica* strains distributed in 2,497 non-pathogenic isolates from lineages 1Aa and 1Ab, and 5,145 specimens belonging to 8 pathogenic lineages. Among pathogenic isolates, lineage 4 was the most common (87.2%) followed by lineages 2/3-9b (10.6%), 2/3-5a (1.2%), 2/3-9a (0.6%), 3-3b, 3-3c, 1B, and 3-3d (0.1% per each). Importantly, we developed a routine surveillance system based on a new typing method consisting of a 1,727-genes core genome Multilocus Sequence Typing (cgMLST) specific to the species *Y. enterocolitica* followed by isolate clustering. Thresholds of allelic distances (AD) were determined and fixed for the clustering of isolates: AD ≤ 5 for lineages 4, 2/3-5a, and 2/3-9a, and AD ≤ 3 for lineage 2/3-9b. Clustering programs were implemented in 2019 in routine surveillance to detect genomic clusters of pathogenic isolates. In total, 419 clusters with at least 2 isolates were identified, representing 2,504 of the 3,503 isolates characterized between 2019 and 2021. Most clusters (*n* = 325) comprised 2 to 5 isolates. The new typing method proved to be useful for the molecular investigation of unusual grouping of cases as well as for the detection of genomic clusters in routine surveillance.

**IMPORTANCE:**

We describe here the new typing method used for molecular surveillance of *Yersinia enterocolitica* infections in France based on a novel core genome Multilocus Sequence Typing (cgMLST) specific to *Y. enterocolitica* species. This method can reliably identify the pathogenic *Y. enterocolitica* subspecies and compare the isolates with a high discriminatory power. Between 2017 and 2021, 5,145 pathogenic isolates belonging to 8 lineages were characterized and lineage 4 was by far the most common followed by lineage 2/3-9b. A clustering program was implemented, and detection thresholds were cross-validated by the molecular and epidemiological investigation of three unusual groups of *Y. enterocolitica* infections. The routine molecular surveillance system has been able to detect genomic clusters, leading to epidemiological investigations.

## INTRODUCTION

*Yersinia enterocolitica* is a well-known pathogen responsible for enteric yersiniosis which is the third most reported zoonosis in humans in Europe (1). Infection in humans is mainly caused by the consumption of undercooked pork meat or other contaminated food (2, 3). After an incubation period of 3 to 7 days, patients may develop a self-limited gastroenteritis characterized by diarrhea, fever, and sometimes abdominal pain mimicking appendicitis (4). Children less than 10 years old are most affected. However, adults may also be infected and patients with underlying conditions such as iron overload, hemochromatosis, diabetes, and cirrhosis may develop severe symptoms, deep abscesses, and systemic infections (5).

*Y. enterocolitica* belongs to the *Yersiniaceae* family (6). The species is divided into six biotypes (BT): 1A, 1B, 2, 3, 4, 5, and in more than 52 serotypes. Only isolates belonging to the biotypes 1B, 2, 3, 4, and 5 hosts the virulence plasmid *pYV* and are considered pathogenic. Pathogenic biotypes 2, 3, 4, and 5 are strongly associated with few O serotypes and form bioserotypes: 2/O:9, 2/O:5,27, 3/O:3, 4/O:3, and 5/O:3, while BT1B may be associated with a wider variety of O serotypes (7). Recent advances in sequencing methods have made core genome Multilocus Sequence Typing (cgMLST) a rapid and reliable approach for the identification of *Yersinia* species and subspecies. The procedure is based on a 500-genes scheme specific to the *Yersinia* genus (8). Using this method, the species *Y. enterocolitica* is divided into 13 lineages with a very good correspondence between lineages and bioserotypes (8). Whereas, BT1B, 4, and 5 groups in unique lineages 1B, 4, and 5, respectively, other biotypes are split in different lineages: (i) BT1A isolates belong to lineages 1Aa and 1Ab; (ii) BT2/O:9 isolates belong to lineages 2/3-9a and 2/3-9b; (iii) BT2/O:5,27 isolates belong to lineages 2/3-5a and 2/3-5b; and (iv) BT3/O:3 isolates belong to lineages 3-3a, 3-3b, 3-3c, and 3-3d.

Human infections due to *Y. enterocolitica* mainly occur as sporadic cases. However, a few outbreaks have been reported worldwide, caused by *Y. enterocolitica* of various bioserotypes such as 4/O:3 in Sweden and Denmark (9), 2/O:9 in Norway (10), and 1B/O:8 in the United States (11). Epidemiological and molecular investigation of outbreaks is crucial as it can lead to the identification of the common source of contamination and remove it from the food chain.

In France, the surveillance of enteric yersiniosis is based on two systems: epidemiological surveillance with the notification of unusual grouping of yersiniosis cases in time and space to public health authorities, and microbiological and molecular surveillance conducted on *Yersinia* isolates by the *Yersinia* National Reference Laboratory (YNRL). Although the notification of enteric yersiniosis is not mandatory in France, *Yersinia* isolates, together with clinical and demographic data, are regularly sent by some medical laboratories to the French YNRL for complete characterization including the identification of the species and the lineage. Since December 2017, genomes of *Yersinia* isolates have been sequenced, and species and sub-species are identified using the 500-genes cgMLST specific to the *Yersinia* genus (8). However, due to its relatively low number of genes, this scheme is not used to detect clusters of closely related isolates. Therefore, we developed a 1,727-core genes scheme specific to the *Y. enterocolitica* species to determine the genetic distances between bacterial isolates with high discriminatory power. Clusters of genetically close isolates can be now notified to Santé publique France, the French public health agency.

Here, we report the molecular surveillance of *Y. enterocolitica* infections for the period 2017–2021. We reveal for the first time the distribution of *Y. enterocolitica* lineages circulating in France. We show that the integrated 1,727-genes cgMLST-based surveillance of *Y. enterocolitica* is useful for the molecular investigation of unusual grouping of *Y. enterocolitica* infections notified by public health authorities and is also able to detect genomic clusters leading to epidemiological investigations.

## MATERIALS AND METHODS

### *Y. enterocolitica* isolates and taxonomic assignment

Clinical isolates of *Y. enterocolitica* (*n* = 7,642) received between 2017 and 2021 were genotypically assigned.

Isolation, genome sequencing, and taxonomic assignment of isolates were performed as described by Savin et al. (8). Briefly, Whole Genome Sequencing (WGS) was conducted with the Illumina technology (NextSeq sequencing machines, Illumina, San Diego, CA, USA) and the 500-genes cgMLST specific to the genus *Yersinia* was carried out using the Bacterial Isolate Genome Sequence Database (BIGSdb) software tool (12, 13) in a database created for the *Yersinia* genus in the Institut Pasteur’s MLST and cgMLST resource https://bigsdb.pasteur.fr/yersinia.

### cgMLST specific to the *Y. enterocolitica* species

A cgMLST specific to the species *Y. enterocolitica* was developed. Core genes definition and selection of the genes specific to *Y. enterocolitica* were performed from a set of 285 genomes as described by Savin et al. for the species *Yersinia pseudotuberculosis* (14) and resulted in 1,727 core genes deemed suitable for cgMLST analysis. A database was created for *Y. enterocolitica* in BIGSdb. Sixty genomes representative of the whole data set diversity of *Y. enterocolitica* were uploaded into the isolates database, and the reference alleles of the 1,727 cgMLST loci were defined in the linked database of reference sequences.

The genome of each isolate identified as pathogenic *Y. enterocolitica* was submitted in routine to this new 1,727-genes *Y. enterocolitica-*cgMLST scheme to determine its allelic profile.

Evaluation of the genetic distance with other isolates from the database was performed using a clustering program implemented in BIGSdb to group isolates with (i) ≤5 allelic differences and (ii) ≤3 allelic differences as the thresholds of allelic distance for considering closely related isolates were adjusted to ≤5 and ≤3 for lineages 4 and 2/3-9b, respectively.

Comparison of the allelic profiles was performed either with an in-house script (Julien Guglielmini, unpublished data) similar to the Genome Comparator plugin implemented in BIGSdb or by the construction of a minimum spanning tree (MST) with GrapeTree (15), using the corresponding BIGSdb plugin.

### Discriminatory power determination

The discriminatory power of the molecular typing method was determined using the Simpson’s Index of diversity. It calculates the probability of a technique to attribute the same profile to epidemiologically unrelated isolates. The higher and closer to 1 the index is, the better the discriminatory power is (16).

### Epidemiological investigations

Cases of *Y. enterocolitica* infection (or their legal representatives) were contacted by Santé publique France and queried about their exposure to animals, visits to natural areas (sea, lake, forest, river, farms), drinking water supply and food consumption (dairy products, delicatessen, meats, eggs, fresh vegetables, unpeeled fruits), using a standard trawling questionnaire. The questionnaire covered the 7 days before the onset of the symptoms.

## RESULTS

### Distribution of *Y. enterocolitica* lineages in France in 2017–2021

The YNRL received 7,642 clinical *Y. enterocolitica* isolates from 2017 to 2021. They came from 7,561 patients distributed in 7,481 patients with 1 isolate, 79 patients with 2 specimens (for 7 of them they were isolated in the same sample), and 1 patient with 3 specimens isolated within 9 days of interval. Isolates were distributed in 2,497 (32.7%) strains belonging to the non-pathogenic lineages 1Aa and 1Ab, and 5,145 (67.3%) specimens belonging to the 8 pathogenic lineages 1B, 2/3-5a, 2/3-9a, 2/3-9b, 3-3b, 3-3c, 3-3d, and 4 (Table 1). There were no isolates from lineages 3-3a, 2/3-5b, nor 5.

**TABLE 1:** Number of *Y. enterocolitica* isolates by lineage and year

Lineage	2017	2018	2019	2020	2021	Total	%
Non-pathogenic							
1Aa	378	504	523	487	562	2,454	98.3
1Ab	4	17	6	8	8	43	1.7
Subtotal	382	521	529	495	570	2,497	100
Pathogenic							
1B	1	0	2	0	2	5	0.1
2/3-5a	16	10	14	5	17	62	1.2
2/3-9a	3	9	7	5	10	34	0.6
2/3-9b	65	78	101	92	208	544	10.6
3-3b	3	2	0	2	1	8	0.1
3-3c	0	1	2	1	2	6	0.1
3-3d	0	1	1	0	0	2	0.1
4	639	814	997	848	1,186	4,484	87.2
Subtotal	727	915	1,124	953	1,426	5,145	100
Total	1,109	1,436	1,653	1,448	1,996	7,642	

The number of pathogenic *Y. enterocolitica* isolates sent to the YNRL nearly doubled in 5 years, growing every year except in 2020. Among the pathogenic isolates, lineage 4 was by far the most frequently identified (87.2%) followed by lineage 2/3-9b (10.6%). The other pathogenic lineages were more rarely identified (Table 1).

**Fig 1:**
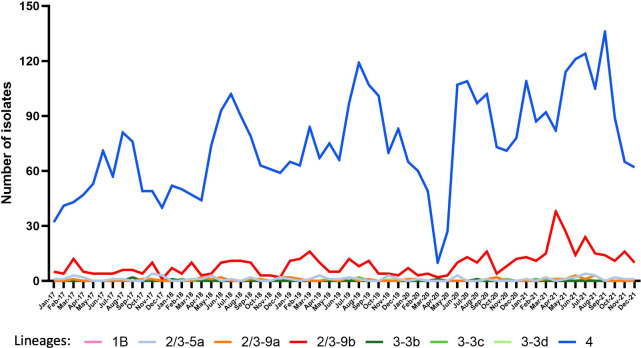
Number of pathogenic *Y. enterocolitica* isolates by lineage and by month and year, France 2017–2021.

The monthly distribution of the pathogenic *Y. enterocolitica* isolates showed a seasonal peak in summer for infections due to lineage 4 (Fig. 1). An unusual pic was observed in the winter of 2020–2021. Lineage 2/3-9b infections occurred without any obvious seasonal pattern. However, a sevenfold increase in 2/3-9b isolates was observed in April and May 2021 compared to 2017–2020 (Fig. 1). Concerning the other lineages, the number of isolates was too low to identify a pattern.

### Genetic relatedness of the *Y. enterocolitica* isolates using a cgMLST specific to *Y. enterocolitica*

**Fig 2:**
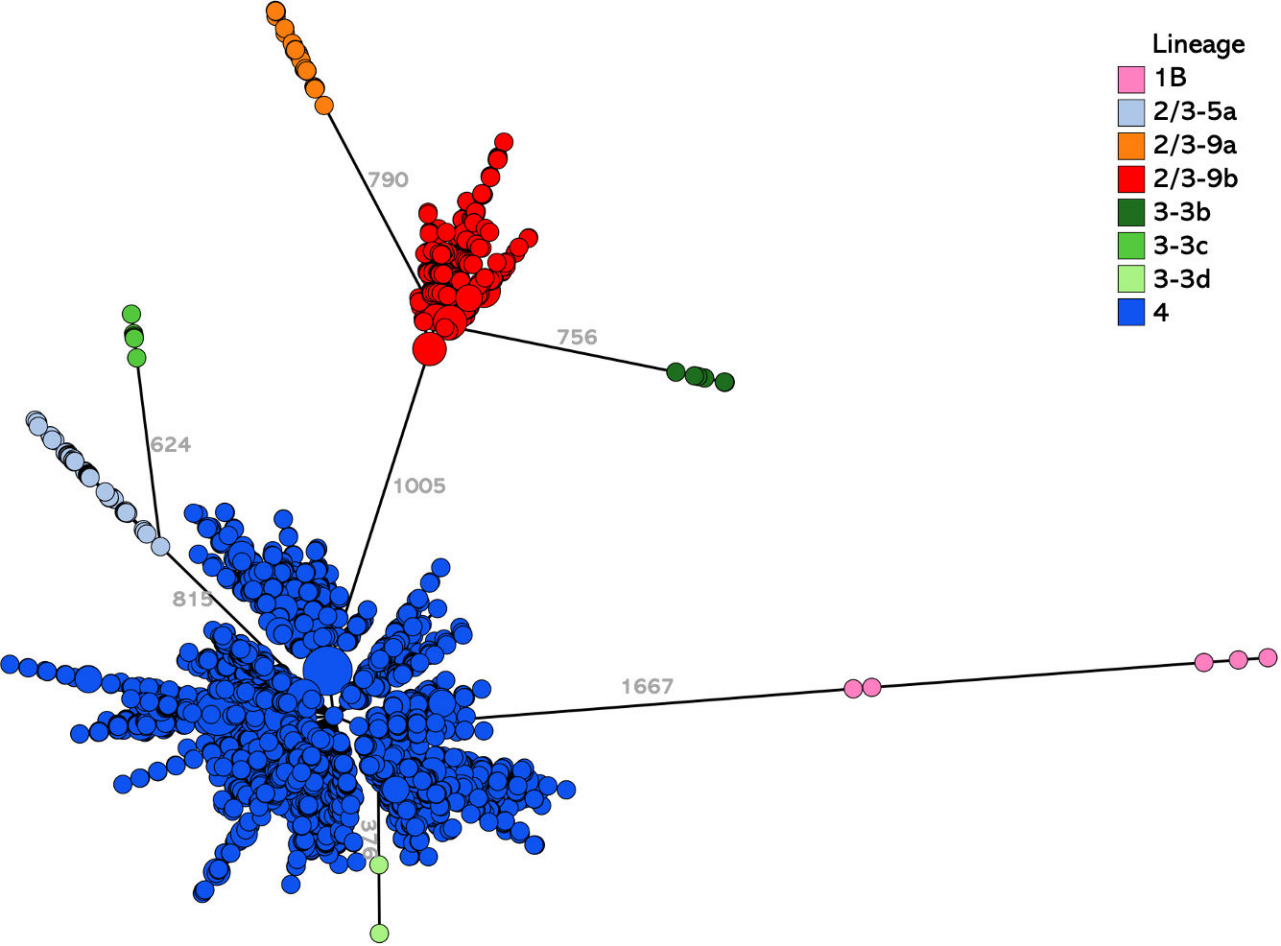
Minimum spanning tree of the 5,145 pathogenic *Y. enterocolitica* isolates, characterized with the 1,727 genes cgMLST at the YNRL, 2017 to 2021. Numbers close to the branches reveal the minimum allele differences between lineages. Circle colors correspond to the isolates’ lineages.

To detect clusters of genetically close isolates, we developed a cgMLST with a 1,727-core genes scheme, specific to the species *Y. enterocolitica*. First, we evaluated the ability of this new cgMLST to discriminate isolates, by analyzing the allelic distances between the isolates of the 8 pathogenic lineages initially identified with the 500-genes *Yersinia*-cgMLST. The matrix of the lowest distances between pathogenic lineages is shown in Table 2 and the lowest distances from the lineages are presented in a minimum spanning tree (Fig. 2). Isolates of the 8 lineages are separated by at least 376 to 1,668 allele differences depending on the lineage. Isolates of lineages 4 and 3-3d are the closest since they are separated by at least 376 allele differences. Isolates of lineage 1B are the farthest from the other lineages. These results confirm that the lineages of *Y. enterocolitica* are well demarcated from each other by the 1,727-genes *Y. enterocolitica*-cgMLST.

**TABLE 2:** Lowest allelic distances between pathogenic *Y. enterocolitica* isolates belonging to the 8 lineages using 1,727-genes cgMLST

	1B	2/3-5a	2/3-9a	2/3-9b	3-3b	3-3c	3-3d	4
1B								
2/3-5a	1,668							
2/3-9a	1,663	934						
2/3-9b	1,666	910	790					
3-3b	1,665	903	758	756				
3-3c	1,668	624	937	893	887			
3-3d	1,662	813	1,023	1,007	1,004	678		
4	1,667	815	1,022	1,005	1,002	677	376	

Then, we analyzed the allelic distances (AD) of the isolates within each lineage to evaluate their genetic relationship. A distance matrix was generated for each population.

**Fig 3:**
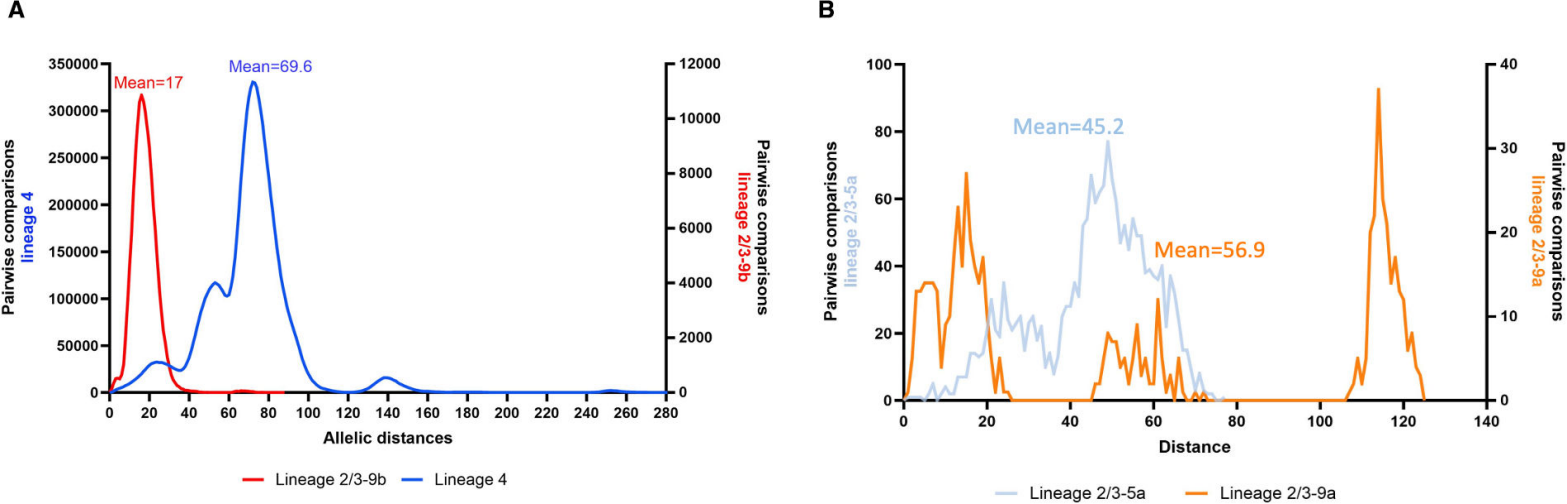
(**A**) Distribution of allelic distances between *Y. enterocolitica* isolates of lineage 4 (blue) and lineage 2/3-9b (red), characterized in 2017–2021. (**B**) Distribution of allelic distances between *Y. enterocolitica* isolates of lineage 2/3-5a (light blue) and lineage 2/3-9a (orange), characterized in 2017–2021.

A pairwise comparison of the 4,484 allelic profiles corresponding to 4,430 unique cgMLST types within lineage 4 showed distances ranging from 0 to 286 with a mean of 69.6 and a standard deviation of 23.7 (Fig. 3A). The graphical distribution of the distances shows 4 peaks and a wide range of distances suggesting a high diversity among the population of lineage 4 isolates. However, the presence of very short AD revealed the circulation of very closely related isolates and suggested that clonal outbreaks probably occur and could be detected (Fig. 3A).

A pairwise comparison of the 544 allelic profiles of lineage 2/3-9b corresponding to 538 unique cgMLST types showed a clonal distribution (Fig. 3A). Allele differences ranged from 0 to 88. Despite a few distances >40, the distribution curve showed a narrow shape with a mean distance of 17 and a standard deviation of 6.5, suggesting a low genetic diversity among the lineage 2/3-9b isolates. As for lineage 4, the presence of short AD means that very close isolates circulated and suggested that outbreaks may occur and could be detected.

AD between the 62 isolates of lineage 2/3-5a ranged from 0 to 77, with a mean of 45.2 and a high standard deviation of 14.3 (Fig. 3B) while AD between the 34 isolates of lineage 2/3-9a ranged from 0 to 124, with a mean of 56.9 and a very high standard deviation of 47.2 suggesting a high diversity among these isolates (Fig. 3B). The presence of short AD between isolates in these 2 lineages also suggests that genetically very close isolates circulated.

Concerning the other lineages, the number of isolates was too low to infer a relevant genetic relationship.

### Threshold distances for considering closely related *Y. enterocolitica* isolates

To identify a threshold distance for considering very closely related isolates of lineage 4, 2/3-9b, 2/3-5a, and 2/3-9a, we analyzed the AD between isolates recovered during family outbreaks and between isolates found within the same patient at different times.

**Fig 4:**
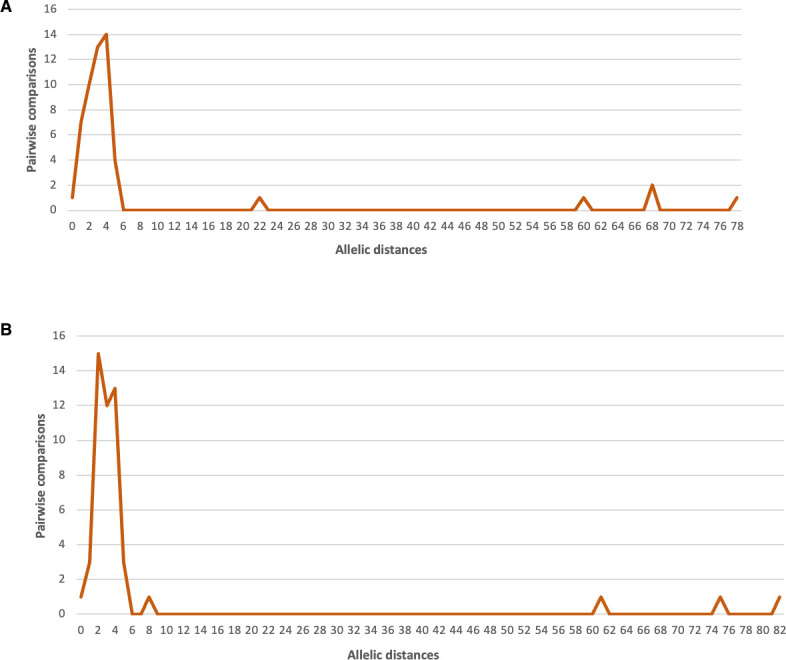
(**A**) Allelic distances between *Y. enterocolitica* isolates of lineage 4 found in members of the same family, using the 1,727-genes cgMLST, over the period 2017–2021. (**B**) Allelic distances between *Y. enterocolitica* isolates of lineage 4 were found in the same patient, using the 1,727-genes cgMLST, over the period 2017–2021.

Concerning lineage 4, we found 50 families with 2 isolates and 2 with 3 isolates (Table 3). The distribution of AD is represented in Fig. 4A. In siblings of 4 families, the AD was equal to or greater than 60, suggesting that the siblings were infected by two different strains, even though the isolation date was the same for the siblings of 3 families. However, in the other families, the AD ranged from 0 to 5 except one distance of 22 with a median of 3.0. We also found 51 patients for which two isolates were identified (Table 4). Sample matrices included stools, blood, and joint fluid, and the time intervals of paired isolates ranged from 0 to 367 days. AD ranged from 0 to 8 except for three pairs of isolates with AD of 61, 75, and 82, suggesting that these three patients have been infected by two different strains (Fig. 4B). As for the family outbreaks, AD showed a median distance of 3.0. The patient with isolates B7 and B8 was a 96-year-old woman, whose first specimen was isolated in the blood during a septicemic episode and whose second specimen was isolated 1 year later in the joint fluid aspirated from a previously reconstructed hip joint. The two isolates were separated by only five allelic differences. The time interval of paired isolates from stools ranged from 1 to 50 days. AD remained ≤5 (except in one case with 8 AD and three cases with >60 AD), regardless of the interval of isolation dates. Of note, the very distant B101 and B102 specimens were isolated from the same patient on the same day, showing that a patient can be infected by two distinct *Y. enterocolitica* lineage 4 strains. The pairwise comparison of closely related isolates from family outbreaks and from the same patients in 2017–2021 showed AD ≤ 5. This value was used as a preliminary threshold for the clustering of *Y. enterocolitica* lineage 4 isolates from 2019. Considering this threshold, Simpson’s index of diversity for the lineage 4 population was estimated to be 1, confirming the excellent discriminatory power of the cgMLST for the typing of *Y. enterocolitica* lineage 4 isolates.

**TABLE 3:** Allelic distances between isolates of lineage 4 found in members of the same family, for 50 families with 2 isolates and 2 families with 3 isolates, using 1,727-genes cgMLST, over the period 2017–2021^*c*^

Code of the isolates	Time interval (days)	Allelic distance
A1-A2^*a*^	0	3
A3-A4^*a*^	0	4
A5-A6^*a*^^,*b*^	0	1
A7-A8^*a*^	0	2
A9-A10^*a*^	0	2
A11-A12	0	1
A13-A14	0	2
A15-A16	0	3
A17-A18	0	3
A19-A20	0	3
A21-A22	0	4
A23-A24	0	4
A25-A26	0	2
A27-A28^*b*^	0	2
A29-A30	0	22
A31-A32	0	60
A33-A34	0	78
A35-A36	0	68
A37-A38^*a*^	1	1
A39-A40^*a*^	1	3
A41-A42^*a*^	1	2
A43-A44^*a*^	1	5
A45-A46	1	4
A47-A48	1	4
A49-A50	1	2
A27-A52^*b*^	1	3
A52-A28^*b*^	1	4
A55-A56	2	1
A57-A58	2	3
A59-A60	2	2
A61-A62	3	2
A63-A64	3	3
A65-A66	4	4
A67-A68^*a*^	5	3
A69-A70	5	3
A71-A72	5	4
A73-A74	6	3
A75-A76	6	0
A77-A78^*a*^	8	5
A79-A80^*a*^	8	4
A81-A82	9	4
A83-A84	9	5
A85-A5^*a*^^,*b*^	11	3
A85-A6^*a*^^,*b*^	11	3
A89-A90	11	4
A91-A92	11	2
A93-A94^*a*^	12	5
A95-A96^*a*^	12	1
A97-A98	12	3
A99-A100^*a*^	14	1
A101-A102	15	1
A103-A104^*a*^	17	1
A105-A106	18	4
A107-A108^*a*^	19	4
A109-A110	31	4
A111-A112	51	68

^*a*^ Both isolates recovered in 2017 or 2018.

^*b*^ Isolates recovered in families with 3 isolates.

^*c*^ The time interval is the number of days between the isolation of the two strains compared two-by-two.

**TABLE 4:** Allelic distances between isolates of lineage 4 found in the same patient, for 51 patients, using 1,727-genes cgMLST, over the period 2017–2021^*b*^

Code of the isolates	Sample matrix	Time interval (days)	Allelic distance
B1-B2	Blood	1	0
B3-B4^*a*^	Blood	3	4
B5-B6^*a*^	Blood	37	2
B7-B8	Blood/joint fluid	367	5
B9-B10^*a*^	Blood/stools	0	2
B11-B12	Stools	1	4
B13-B14	Stools	2	3
B15-B16	Stools	2	3
B17-B18^*a*^	Stools	3	2
B19-B20^*a*^	Stools	3	3
B21-B22	Stools	3	3
B23-B24	Stools	3	1
B25-B26	Stools	3	3
B27-B28	Stools	3	1
B29-B30	Stools	3	3
B31-B32	Stools	4	4
B33-B34	Stools	5	2
B35-B36	Stools	5	4
B37-B38	Stools	7	2
B39-B40	Stools	7	2
B41-B42	Stools	7	3
B43-B44	Stools	7	4
B45-B46	Stools	11	3
B47-B48	Stools	11	2
B49-B50	Stools	11	2
B51-B52	Stools	12	2
B53-B54	Stools	12	2
B55-B56^*a*^	Stools	14	3
B57-B58^*a*^	Stools	14	4
B59-B60	Stools	15	4
B61-B62^*a*^	Stools	16	2
B63-B64	Stools	16	2
B65-B66	Stools	17	3
B67-B68	Stools	17	5
B69-B70	Stools	17	3
B71-B72	Stools	21	2
B73-B74	Stools	22	8
B75-B76	Stools	23	4
B77-B78	Stools	25	4
B79-B80^*a*^	Stools	26	4
B81-B82^*a*^	Stools	26	5
B83-B84	Stools	29	1
B85-B86^*a*^	Stools	30	3
B87-B88	Stools	30	4
B89-B90	Stools	34	4
B91-B92	Stools	40	4
B93-B94	Stools	44	2
B95-B96^*a*^	Stools	50	2
B97-B98	Stools	27	61
B99-B100	Stools	3	75
B101-B102	Stools	0	82

^*a*^ Both isolates recovered in 2017 or 2018.

^*b*^ The time interval is the number of days between the isolation of the two strains compared two-by-two.

Concerning the lineage 2/3-9b, we found 2 families in which 2 isolates were identified: isolates were separated by 2 AD. We also identified 3 patients with pairs of isolates: one pair with a 3 AD was collected in the same patient from blood and joint fluid on the same day, the 2 other pairs were recovered from stools at 8 and 10 days of intervals, with 5 and 3 AD, respectively. The threshold distance for related isolates during outbreaks was determined considering the lower genetic diversity of the lineage 2/3-9b (mean AD = 17) population compared to lineage 4 (mean AD = 69.6), and the AD observed in the same families and same patients. As we did not find any isolates from the same patient or the same family in 2017–2018, we found 4 pairs of isolates from the same family earlier in 2016 with 3, 3, 2, and 5 AD. We also found 2 pairs of isolates from the same patient in 2016 with 0 and 3 AD. Thus, we fixed a preliminary threshold of 3 AD for the clustering of *Y. enterocolitica* lineage 2/3-9b isolates and used it from 2019. Considering this threshold, the Simpson’s index of diversity for the lineage 2/3-9b population was also estimated to be 1, confirming the excellent discriminatory power of the cgMLST for the typing of *Y. enterocolitica* lineage 2/3-9b isolates.

Regarding the lineage 2/3-5a, AD between 2 isolates collected during a family outbreak and between a pair of isolates from the same patient at a 4-day interval were 1 and 3, respectively. Concerning the lineage 2/3-9a, a 3-allele difference was found between 2 isolates recovered in the same family. Based on both these values and the high genetic diversity of the *Y. enterocolitica* 2/3-5a and 2/3-9a populations, an AD ≤5 was fixed for considering genetically very close isolates of *Y. enterocolitica* 2/3-5a and 2/3-9a.

### Use of epidemiological investigations to corroborate distance thresholds

Three unusual groups of enteric yersiniosis cases were notified to Santé publique France between 2019 and 2021.

At the end of December 2019, a medical laboratory in the Auvergne-Rhône-Alpes region alerted the Regional Health Authority and Santé publique France of an unusual pattern of enteric yersiniosis. The laboratory isolated *Y. enterocolitica* in the stools of 7 patients living in 2 nearby villages, with symptoms over the same week. In total six children (2 to 15 years old) and one adult (21 years old) were identified. The male-to-female sex ratio was 0.75 (4 females and 3 males).

The YNRL received and characterized the 7 *Y. enterocolitica* isolates, one per patient. They were identified as belonging to lineage 4 and were submitted to the *Y. enterocolitica* 1,727 genes cgMLST. AD between 6 isolates was less than 5, while a 7th isolate (coded C4) was much more distant from the others (AD from 64 to 67) (Table 5). The 5-mismatch clustering program grouped the 6 close isolates in one cluster, consisting of these 6 isolates only. An additional isolate, recovered from a 2-year-old child living in the same geographical area, was sent by the same laboratory in January 2020 and belonged to the same cluster (2 to 3 AD from the other isolates). An investigation was initiated by Santé publique France and 5 cases of the cluster could be interviewed. Two cases were siblings, and two other children had the same childminder. No common exposure was identified among the 5 cases (no common food item, place of purchase, brand, product, restaurant). The patients resided in areas provided by the same water distribution network. Although no dysfunction of the water treatment plant was identified, it was noted that the time of journey of water in the reservoir was long. Finally, a hydric hypothesis of contamination was suggested but could not be confirmed.

**TABLE 5:** Distance matrix of isolates found in Auvergne-Rhône-Alpes in December 2019

	C1	C2	C3	C4	C5	C6	C7	C8
C1	0							
C2	3	0						
C3	4	2	0					
C4	66	65	67	0				
C5	3	1	3	64	0			
C6	4	2	2	65	1	0		
C7	4	2	2	65	1	0	0	
C8	3	2	3	66	3	3	3	0

In June 2020, a school doctor alerted the Regional Health Authority of Nouvelle-Aquitaine region of 10 pupils in the same school who developed gastroenteritis symptoms over a week. In total, eight pupils had stool samples taken, with the identification of *Y. enterocolitica*. The investigation conducted by the Regional Health Authority revealed a single meal common to all cases (radish, cooked ham, broccoli, soft white cheese). There were no remaining food items to sample to evaluate a microbiological link with the clinical isolates. The YNRL characterized the eight isolates. They belonged to lineage 4 and were grouped in a cluster with the 5-mismatch clustering criteria. AD between the eight isolates ranged from 1 to 5 (Table 6). The genomic cluster contained 32 strains, isolated from July 2019 to July 2020, from four regions. Three other specimens were isolated in the same week from the same region, but no epidemiological link could be found between them and the school cases.

**TABLE 6:** Distance matrix of isolates found for eight patients in Nouvelle-Aquitaine in June 2020

	D1	D2	D3	D4	D5	D6	D7	D8
D1	0							
D2	2	0						
D3	4	3	0					
D4	4	3	3	0				
D5	2	2	2	2	0			
D6	3	3	1	3	1	0		
D7	5	3	2	2	3	2	0	
D8	4	4	2	4	2	1	3	0

In February 2021, the Regional Health Authority of Provence-Alpes-Côte d’Azur region investigated four cases of enteric yersiniosis among young children from the same geographical area, who had experienced gastrointestinal symptoms between 2 January and 1 February and had stool samples taken and confirmed positive for *Y. enterocolitica*. The investigation revealed that the four children were in different childcare centers provided by the same central kitchen. The central kitchen was visited by hygiene teams, and food and environmental samples were taken. *Y. enterocolitica* was isolated from grated carrots consumed by the cases. The YNRL received and characterized the clinical isolates: they were all lineage 4 but only two were close isolates with 2 AD, the others were >90 AD from one another. The *Y. enterocolitica* isolated from the grated carrots was characterized as lineage 1Aa by the YNRL.

These investigations supported the AD threshold previously fixed to 5 for considering closely related isolates of *Y. enterocolitica* lineage 4 and showed that the 1,727-genes *Y. enterocolitica*-cgMLST was useful to consider isolates as unrelated, allowing the epidemiologists to focus their investigations only on related cases.

### Clusters detected by the routine molecular surveillance system

To detect outbreaks of *Y. enterocolitica* infections, a routine surveillance procedure was implemented in 2019 at the YNRL consisting of the monthly detection of isolates belonging to the same cluster determined by cgMLST. Using a 5-mismatch clustering for lineages 4, 2/3-9a, and 2/3-5a, and a 3-mismatch clustering for lineage 2/3-9b as defined above, 419 clusters were detected between 2019 and 2021. These 419 clusters included 2,504 of the 3,503 isolates characterized (71.5%). Out of them, 363 clusters belonged to lineage 4, followed by 48, 4, and 4 belonging to the lineages 2/3-9b, 2/3-5a, and 2/3-9a, respectively. Most clusters (325/419) comprised 2 to 5 isolates while 52 clusters comprised 6 to 10 isolates and 42 clusters had more than 10 isolates. Only 210 clusters were notified to Santé publique France (those with less than 6 weeks between the two most recent isolates).

In April 2021, the YNRL notified to Santé publique France of a cluster of 9 specimens of lineage 2/3-9b isolated between 27 March and 11 April. Given the lineage 2/3-9b identified, less frequent and associated with more severe infections in the literature, and the unusually high number of cases included in the cluster (no 2/3-9b cluster of more than 2 isolates since 2017), Santé publique France initiated an epidemiological investigation to identify any common exposures indicating a possible common source of contamination and to implement control measures. In total, 44 cases were identified by the YNRL in this genomic cluster with isolation dates between 27 March and 16 May. The 44 cases lived in 11 regions of France, with 24 of them in two regions (Bourgogne Franche Comté and Occitanie). The male-to-female sex ratio was 1.1 (21 females and 23 males). Age ranged from 2 to 100 years (median: 35 years). Specimens were mainly isolated from stools (43/44), but one was isolated from blood in a patient hospitalized and receiving hemodialysis.

Santé publique France successfully interviewed 34 cases. Symptoms onsets ranged from 10 March to 29 April. Of the 34 cases, 30 had consumed beef meat and 29 had consumed leafy salad, although no commonplace of purchase or brand stood out. No other food item consumed by at least 50% of the cases was identified.

**Fig 5:**
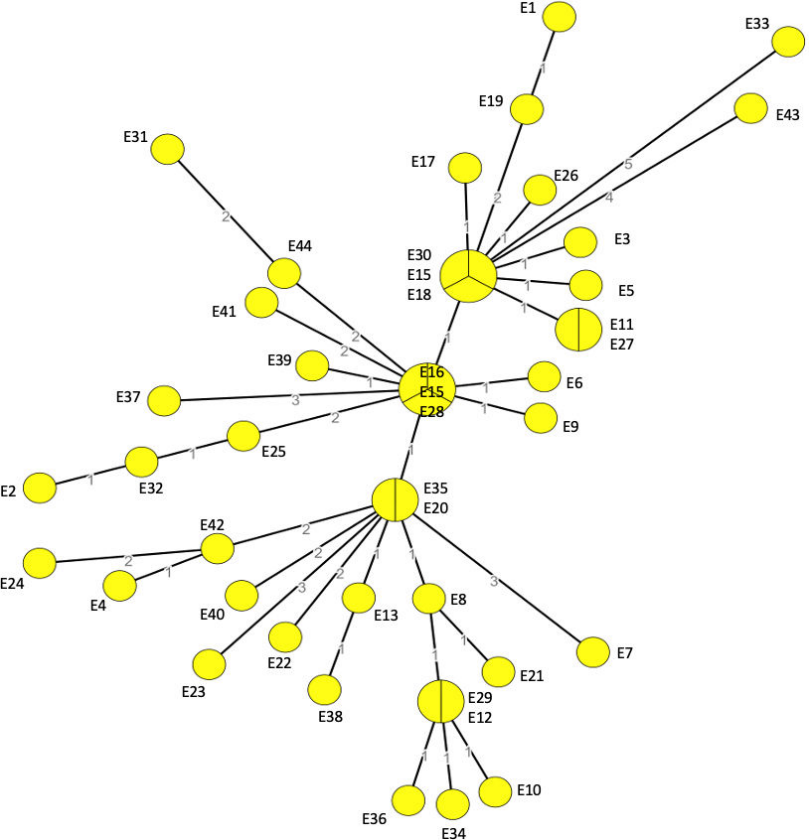
Minimum spanning tree obtained from the allelic profiles of the cgMLST (1,727 genes) on the 44 *Y. enterocolitica* 2/3-9b isolates belonging to the same cluster. Numbers close to branches reveal allele differences. The pie chart identifies several isolates with the same allelic profile.

The molecular analysis of the 44 isolates by the YNRL found that the average distance and the standard deviation were 3.3 ± 1.3. The lowest distances within the isolates are presented in an MST generated by GrapeTree (Fig. 5). These short distances confirmed their close genetic relationship.

These results confirm that the routine surveillance system based on the 1,727-genes *Y. enterocolitica*-cgMLST can detect clusters, which must be corroborated by epidemiological investigations to identify a common source of contamination.

## DISCUSSION

Between 2017 and 2021, the number of isolates of *Y. enterocolitica* reported to the YNLR increased every year, except in 2020, impacted by the COVID-19 pandemic. A regular increase has already been observed from 2012 to 2021 with 314 and 1,451 reported cases, respectively (17, 18). This increase is probably related to the use of mass spectrometry for bacterial identification in clinical laboratories. This automated, quick, and easy method can analyze numerous suspected colonies in the same run which increases the probability of identifying a *Yersinia* spp. Moreover, since its implementation in medical laboratories in 2017 in France, the screening by PCR multiplex of enteric pathogens has led to a culture of the stools detected positive, which improves the isolation rate of *Yersinia* spp. (19). The *Y. enterocolitica* isolates identified by medical laboratories must be further characterized by the YNRL to exclude non-pathogenic lineages for clinical diagnosis, thus explaining the increase of isolates sent to the YNRL.

Species and lineages of the 7,642 *Y. enterocolitica* isolates were successfully identified by the recently developed 500-genes cgMLST specific to the *Yersinia* genus (8). This genotypic characterization is more reliable than the classical phenotypic method consisting of biotyping and serotyping because biotyping may be difficult in case of atypical characteristics and serotyping may be hampered by lack of antisera or by auto agglutinable or non-serotypeable isolates (8).

Non-pathogenic lineages 1Aa and 1Ab, corresponding to the biotype 1A, represented 32% of the *Y. enterocolitica* isolates received at the YNRL. Despite few studies showing toxicity in suckling mice (20), invasion of intestinal cells (21), and virulence in a *Galleria mellonella* infection model (22), the isolates of these lineages are considered non-pathogenic because they lack adhesins, invasin, and the *Yersinia* Outer Proteins which are the main virulence factors in *Y. enterocolitica*. They harbor the *ystB* gene encoding the *Yersinia*-stable toxin B which can cause an accumulation of fluids in the intestines of infant mice, however, there is no evidence of its production in humans (23, 24). Identification of *Yersinia* isolates of these non-pathogenic lineages in symptomatic patients remains important because it leads to the search for other pathogens responsible for the symptoms and it may result in treatment changes.

Eight pathogenic lineages were identified among the clinical *Y. enterocolitica* isolates circulating in France. Lineage 4 was most frequent (87.2%) followed by lineage 2/3-9b (10.6%). Since lineages 4 and 2/3-9b correspond to bioserotype 4/O:3 and 2/O:9, respectively, this is consistent with the figures reported for Europe in 2021 with the most reported bioserotypes being 4/O:3 (83.2%) and 2/O:9 (15.3%) (18). In France, a seasonal peak in summer was observed only with infections due to lineage 4 while lineage 2/3-9b infections occurred without an obvious seasonal pattern. This suggests differences in animal reservoirs and sources of infection. The well-known animal reservoir for the lineage 4 isolates is the pig and it was confirmed in France by a prevalence study in 2010 that found that 74.3% of the pig batches at slaughter were positive for pathogenic *Y. enterocolitica* with 92% of isolates belonging to biotype 4 (25). This increase in summer for the lineage 4 cases might be explained by an increase in barbecues during the summer, with greater consumption of barbecued sausages, chipolatas, ribs, and other pork meat products. The animal reservoir for lineage 2/3-9b is not yet well established. However, a strong association between biotype 2 and bovines, sheep, and goats, was highlighted in a study on the potential sources of enteric yersiniosis in France (26). The number of 2/3-9b isolates sent to the YNRL in 2021 was high compared to previous years, and the genomic cluster investigated in April 2021 could not explain the excess. It is important to continue the surveillance of this lineage to be able to identify any increase in circulation (27).

Regarding lineages 2/3-5a and 2/3-9a, only 17 and 10 isolates, respectively, were identified in 2021 suggesting a low exposure to their reservoirs or a lower circulation of these lineages. Lineages 2/3-5a and 2/3-9a correspond to bioserotypes 2-3/O:5,27 and 2/O:9, which have already been reported in humans and animals in France (26). However, since bioserotype 2/O:9 is divided into two lineages (2/3-9a and 2/3-9b), it could be interesting to identify whether these two lineages have specific reservoirs. Isolates belonging to the other pathogenic lineages 1B, 3-3b, 3-3c, and 3-3d are in a very low number which suggests that the French population is seldom exposed to these lineages. Biotype 1B is known to have caused outbreaks in the United States in the four last decades (11, 28, 29) while biotype 3 was shown predominant in several regions of China (30, 31).

Our study showed that the 1,727-genes *Y. enterocolitica*-cgMLST has an excellent discriminatory power to delineate distinct clusters of very closely related isolates within the different *Y. enterocolitica* lineages. Indeed, the Simpson’s index of diversity is very high, close to 1, for both 2/3-9b and 4 lineages with 3 and 5 AD, respectively, used to define clusters. This high discriminatory power is due to the high number of loci analyzed in cgMLST. Another cgMLST specific to *Y. pseudotuberculosis* with a 1,921 genes scheme was developed at the same time and was able to detect a cluster of clonal *Y. pseudotuberculosis* infection in Corsica in 2020 (14). These cgMLSTs specific to enteropathogenic *Yersinia* species can discriminate much better than other molecular methods such as Pulsed Field Gel Electrophoresis and Multiple Locus Variable tandem repeat Analysis which were used previously to investigate outbreaks (32).

Usually, the genetic distances to determine the similarity between isolates are derived from previous outbreak investigations. Here, the thresholds were primarily established based on family outbreak studies and genetic changes of isolates in patients.

Persistent *Y. enterocolitica* infections have already been reported with late complications such as deep abscesses and arthritis (33), but the persistence of the bacteria in stools was not studied. Here, the pairwise comparison of isolates found within an interval of up to 50 days in the same patients showed that the AD remained small, suggesting that *Y. enterocolitica* may persist in the intestines and be excreted for at least 7 weeks and without important genetic changes. This means that the infection due to *Y. enterocolitica* is not always self-limiting and that the confirmation of the clearance of the bacteria may be relevant in patients with underlying conditions to prevent severe complications. Closely related isolates (5 AD) of *Y. enterocolitica* lineage 4 were also found in a patient presenting with chronic hip arthritis, first in blood and 367 days later in joint fluid. The hip joint reconstruction occurred before the bacteremia episode, suggesting that a bacterial biofilm formed on the biomaterial and bacteria were released from the biofilm leading to the bacteremia episode (34). Infections due to biomaterial-related biofilms are severe complications of arthroplasties. Bacteria attach to the implanted prostheses as well as the periprosthetic tissue and form a biofilm in which they may adopt a slow-growing status (35). This may explain the proximity of the 1-year-separated isolates in blood and joint fluid.

The threshold distance (AD ≤ 5) for considering closely related isolates of lineage 4 has been cross-validated by the molecular and epidemiological investigation of three unusual groups of yersiniosis cases notified by clinicians or microbiologists in the field. The closely related isolates were ≤5 AD from each other while the unrelated isolates were more than 64 AD distant. The 5-mismatch clustering program allowed the clustering of the closely related isolates and excluded the unrelated isolates.

The routine molecular surveillance system of isolates of lineages 4, 2/3-9a, and 2/3-5a is based on a 5-mismatch clustering program. However, as clusters grow, they include more and more isolates which are always ≤5 AD from one isolate but may be more distant from several isolates, and within a large cluster, isolates may group in subclusters. Therefore, it is important to keep in mind the AD between each couple of isolates of the same cluster to interpret their relatedness. Moreover, clusters must be continuously confronted with epidemiological data obtained through outbreak investigations.

The surveillance system for *Y. enterocolitica* lineage 2/3-9b is based on a 3-mismatch clustering program because the population of lineage 2/3-9b circulating in France is more clonal than the population of lineage 4. Despite this lower threshold, the surveillance system detected a large cluster of *Y. enterocolitica* lineage 2/3-9b infections in Spring 2021, which was investigated by Santé publique France. Although the specific food item responsible for the outbreak could not be identified, the investigation highlighted the two foods frequently consumed by the cases before the onset of their symptoms: beef meat and leafy salad. Consumption of beef meat was never reported as a source of human contamination but the bioserotype BT2/O:9 is considered strongly associated with bovines in France (26), suggesting that contaminated beef meat could also be a source of human contamination. However, the two outbreaks of *Y. enterocolitica* BT2/O:9, reported in Norway in 2011 and 2014, were associated with the consumption of contaminated mixed salad and included isolates from geographically disparate areas (10, 36).

It is interesting to note that 71% of isolates characterized in 2019–2021 belonged to genomic clusters. A thorough analysis of the clusters identified, based on time and space distribution and demographic characteristics of the cases, would be interesting for a better understanding of the circulation of the different lineages. This novel 1,727 genes cgMLST is also an excellent tool for assessing the genetic diversity of *Y. enterocolitica* of animal origin and studying their contribution to human enteric yersiniosis.

In conclusion, our study shows that the reporting rate of enteric yersiniosis increases every year in France where 8 pathogenic lineages of *Y. enterocolitica* are currently circulating. Enteric yersiniosis is mainly due to lineage 4 followed by lineage 2/3-9b. A seasonal pattern is observed in infections due to lineage 4. The newly developed 1,727-genes cgMLST specific to *Y. enterocolitica* is a high-resolution typing method that is useful for the molecular investigation of unusual grouping of yersiniosis cases as well as for the detection of genomic clusters in the routine surveillance system. This 1,727-genes *Y. enterocolitica*-cgMLST is available for the scientific community: https://bigsdb.pasteur.fr/yersinia.
